# Histological and contractile changes in the genioglossus muscle after nasal obstruction in growing rats

**DOI:** 10.1038/s41598-023-32921-w

**Published:** 2023-04-17

**Authors:** Karin Harumi Uchima Koecklin, Chiho Kato, Yasunori Abe, Tadachika Yabushita, Satoshi Kokai, Takashi Ono

**Affiliations:** 1 School of Dentistry, Universidad Científica del Sur, Lima, Peru; 2 Department of Orthodontic Science, Graduate School of Medical and Dental Sciences, Tokyo Medical and Dental University (TMDU), Tokyo, Japan; 3 Miyamae Orthodontics, Kanagawa, Japan

**Keywords:** Musculoskeletal system, Oral anatomy, Respiration

## Abstract

The aim of the study was to address the genioglossus muscle physiological and histological changes after unilateral nasal obstruction in growing rats. Fifty-four 6-day-old male Wistar albino rats were randomly divided into control (n = 27) and experimental (n = 27) groups. Unilateral nasal obstruction was performed at 8 days old. Contractile properties of the genioglossus whole muscle were measured at 5-, 7- and 9-week-old, including the twitch and tetanic forces, contraction time, half-decay time, and fatigue index. The histological characteristics of the genioglossus were also evaluated at 5-, 7- and 9-week-old, analyzing the myosin heavy chain composition of the slow, fast, IIa and IIb muscle fiber type, by measuring the number, rate, diameter and cross-sectional area. The maximal twitch force, and tetanic force at 60 Hz and 80 Hz force was significantly increased at all ages after nasal obstruction. The fatigue index was decreased at 5 weeks-old after nasal obstruction. The diameter and cross-sectional area of the fast, IIa and IIb muscle fiber types were increased at 7 and 9 weeks after nasal obstruction, while only the diameter of IIa type and cross-sectional area of IIb type were increased at 5 weeks-old after nasal obstruction. Nasal obstruction during growth affects the whole genioglossus muscle contractile properties and histological characteristics, increasing its force, the diameter and area of its muscle fibers. These changes in the genioglossus muscle may affect the normal growth, development and function of the craniofacial complex.

## Introduction

The tongue comprises both intrinsic and extrinsic muscles that work independently to allow changes in its stiffness, shape and position for proper respiration, mastication, swallowing and speech^1,2^. The genioglossus muscle (GG), an extrinsic muscle of the tongue, is the major tongue-protruding muscle, playing an important role during respiration, helping in the maintenance of the airway flow by keeping the diameter of the upper airway^3,4^. GG activity may be influenced by changes in the normal breathing pattern, such as nasal obstruction and/or mouth breathing. There is also an increase in the electromyographic activity of GG when after nasal obstruction^5^.

Among the different respiratory problems, the tongue play an important role, such as in obstructive sleep apnea and mouth breathing^6,7^, and may also be involved in other orofacial pathologies such as sleep bruxism^8^. Moreover, nasal obstruction has also been associated with The evaluation of tongue function is considered relevant for the diagnosis of these pathologies^8–10^, as it is an important target during alternative treatments such as the myofunctional therapy (MFT). MFT is considered an adjunct treatment in patients with orofacial function problems, such as mouth breathers, and in patients with obstructive sleep apnea, that consist in muscle training, targeting the orofacial muscles, such as the tongue muscles^11,12^. Considering this treatment approach, it is important to understand the muscle contractile and histological changes in conditions such as nasal obstruction, in order to improve the patient’s outcome.

Our previous studies showed an increase in the tongue-protruding forces after unilateral nasal obstruction in growing rats, by changing the muscle contractile properties, while oxygen saturation levels were also lower after nasal obstruction^13,14^. It has been suggested that the muscle force and contractile properties are affected by the cross-sectional area of muscle fiber^15^.

The muscle fibers can be classified as slow-type and fast-type, identifying them by their myosin-heavy chain (MHC) composition^16^. The MHC fiber-type composition is related to the contractile characteristics of the muscle^17,18^. These heavy chains determine the speed of muscle shortening, as myosin is the most important part for muscle contraction^16^. Based on the MHC composition, type I isoform is present in slow-twitch muscle fibers, and type II is present in fast-twitch muscle fibers, while IIa, IIb and IIx type are different fast-twitch isoform types^16^. GG has predominantly fast-twitch type muscle fibers that permit its complex movements^17^.

The aim of this study was to evaluate the histological and contractile changes of GG after unilateral nasal obstruction in growing rats. We hypothesized that nasal obstruction in growing rats affects the contractile properties of GG muscle and alter its muscle fibers.

## Methods

### Animal preparation

**Figure 1:**
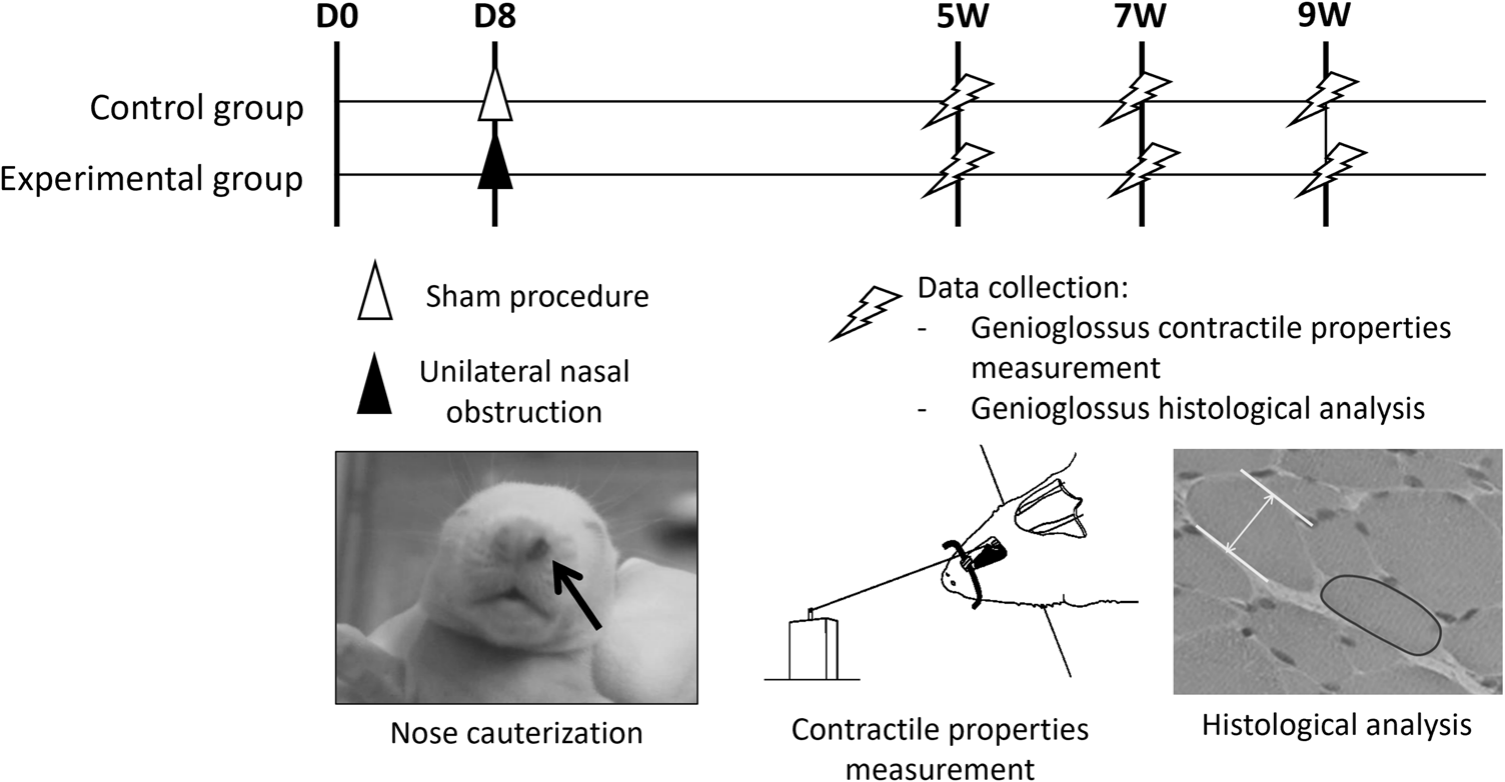
Organigram of experiment procedures. A sham procedure was performed at 8 days old in the control group, while a unilateral nasal obstruction by cauterization of the left nostril was performed in the experimental (nose obstruction) group at 8 days old. Data collection for the contractile properties of the genioglossus muscle, and histological analysis was performed at 5-, 7- and 9-week-old animals.

Fifty-four 6-day-old male Wistar albino rats were divided randomly into control (n = 27) and nose obstruction (n = 27) groups. The organigram of the experimental procedures is shown in Fig. 1. At 8 days-old, rats were anesthetized by hypothermia. This procedure was conducted by placing the pups inside a cold chamber (− 18 °C) for 10 min. Unilateral nasal obstruction was performed by cauterization of the left external nostril^14,19^, by burning the surrounding tissues of the left nostril with a self-constructed cauterization instrument (1 mm in diameter, stainless-steel wire, Tomy Company, Ltd., Tokyo, Japan), occluding the nostril without any chemical or mechanical damage to the olfactory mucosa. The tissue was coated with 3% chlortetracycline (Aureomycin^®^ Ointment; Pola Pharma, Tokyo, Japan) after cauterization to prevent any infection. The pups were placed in a warm blanket (37 °C) for 30 min for recovery and then returned to their mothers.

The control group underwent a sham procedure that involved the placement of the cauterizing instrument 1–2 mm above the left nostril. The rats were checked every week to verify the closure of the cauterized nostril, and if reopening occurred, the pup was not considered for further experiments.

### Nerve stimulation and recording of contraction properties

After nasal obstruction, the rats were divided in 3 different subgroups, to collect the data at 3 different age stages. Data collection took place at 5, 7, and 9 weeks old (n = 7 for each age group, respectively). The rats were anesthetized with ketamine 70 mg/kg and xylazine 7 mg/kg (intraperitoneal injection), and later the hypoglossal nerves were exposed bilaterally^20^, with an incision in the middle of the anterior part of the neck, and bifurcation of the hypoglossal nerve was exposed as previously^13,14^. Following the isolation of the hypoglossal nerve, a 2- to 5-mm section of the lateral branches were removed bilaterally^13,14^.

To record the GG whole-muscle contractile properties, the osseous insertion of GG was detached, and the tendon was sutured with a 20-cm-long 3-0 black silk suture (Matsuda Sutures, Tokyo, Japan). The dorsal part of the tongue was then attached to the palatal vault by cyanoacrylate in order to obtain stability of the tongue, as previously reported^17^.

The GG muscle contraction characteristics measured were the following: twitch force (Tw), contraction time (CT), half-decay time (HDT), fatigue index (FI), tetanic force at 60 Hz (Tet60), and tetanic force at 80 Hz (Tet80), as our previous studies^13,14^. Tw was considered as the maximum tension following a single supramaximal stimulation (1.5 times of maximal stimulation) of the nerve. Both Tet60 and Tet80 represented the force generated after repeated stimulations before complete muscle relaxation^16^. The FI was calculated by the reduction of force following 2 min of stimulation divided by the initial force^21^. CT and HDT were measured from Tw, where CT represented the duration between the onset of the stimulation and the point of 50% peak force, and HDT was considered the time between the onset of stimulation and the point of 50% decay from peak force.

To measure these parameters, the suture previously attached to the tendon of the GG was connected to an isometric force transducer (MLT0420; ADInstruments, Dunedin, New Zealand). The head of the animal fixed onto a platform and the tongue extended at a preloaded force of approximately 3 g, as described previously^20^. Electrical stimulation was supplied from a stimulator (SEN-7203, Nihon Kohden Corporation, Tokyo, Japan) with two tungsten electrodes (0.010″, 250-μm-diameter, epoxylite insulation, 9 MΩ of Impedance measured at 1000 Hz; FHC, Inc., Bowdoin, ME, USA) on the bilateral medial branches of the hypoglossal nerves applied by 0.1-ms rectangular pulses at a supramaximal current (around 500 μA), as described in previous studies^20,22^. Both electrodes were placed simultaneously, with the tip inserted into the medial branches proximal to the bifurcation of the hypoglossal nerve, with one electrode on each side. Stimuli for Tw were delivered at 1 Hz. Stimuli for Tet60 and Tet80 were at 60 and 80 Hz respectively, for 200-ms trains. Stimuli for FI a 100-Hz train for 500 ms.

### Immunohistochemistry of the GG

Histological samples were prepared to examine the MHC muscle fiber composition from all groups at all ages (n = 2). Antibody test exams were performed in each specimen, before the final preparation for MHC analysis was done. After isolating the GG surgically, they were placed in tissue-embedding medium, and paraffin blocks were prepared.

The GG consists of horizontal and oblique fibers that act in the protrusion and depression of the tongue, respectively^23^. In order to observe the horizontal fibers, serial sections of 10-µm thick were cut from the sagittal plane, 1 mm away of the midline, and treated with monoclonal antibodies specific to rat MHC-slow (1:100 dilution, Sigma-Aldrich Co, Saint Louis, Missouri, USA), MHC-fast (1:500 dilution, Sigma-Aldrich Co, Saint Louis, Missouri, USA), MHC-IIa (1:100 dilution, Abcam, Cambridge, Massachusetts, USA), and MHC-IIb (1:100 dilution, Proteintech Group, Rosemont, Illinois, USA) isoforms, and the MHC-fast antibody reacts to all fast type fibers, including the IIa, IIb and IIx type. Sections were embedded with normal blocking serum from bovine. Incubation took place at 4 °C overnight. After washing with PBS, Histofine simple stain MAX-PO (Nichirei Biosciences Inc, Chuo, Tokyo, Japan) was applied. For staining, diaminobenzidine was used.

Fibers were measures from the anterior, middle and posterior part of GG. Muscle fibers were visualized with a Nikon Eclipse 80i microscope (Nikon Instruments Inc, Minato, Tokyo, Japan) with 10× and 20× magnification. The diameter, the cross-sectional area, and percentage of muscle fibers were examined as previous studies^17,18^, using the NIS-Elements Documentation (Nikon Instruments Inc, Minato, Tokyo, Japan).

### Statistical analysis

All data of the contractile properties, diameter, and cross-sectional area is expressed as mean ± standard deviation (SD). A repeated-measures multivariate analysis of variance was performed for intergroup and intragroup statistical comparisons. A simple main-effects analysis with Sidak adjustment was performed for multiple comparisons. Statistical analysis was performed with SPSS Statistics for Windows, Version 13.0J (SPSS Inc., Chicago, IL, USA), with a statistical significance established at *p* < 0.05.

### Ethical approval

The experimental procedures described were approved by the Institutional Animal Care and Use Committee of Tokyo Medical and Dental University (TMDU) (#0160165A and #0170251A). The Principles of laboratory animal care were followed, the Japanese Law on animal protection and the Animal Care Standards of TMDU were followed. All the methods reported followed the ARRIVE guidelines.

## Results

### Contractile properties of the GG

**Figure 2:**
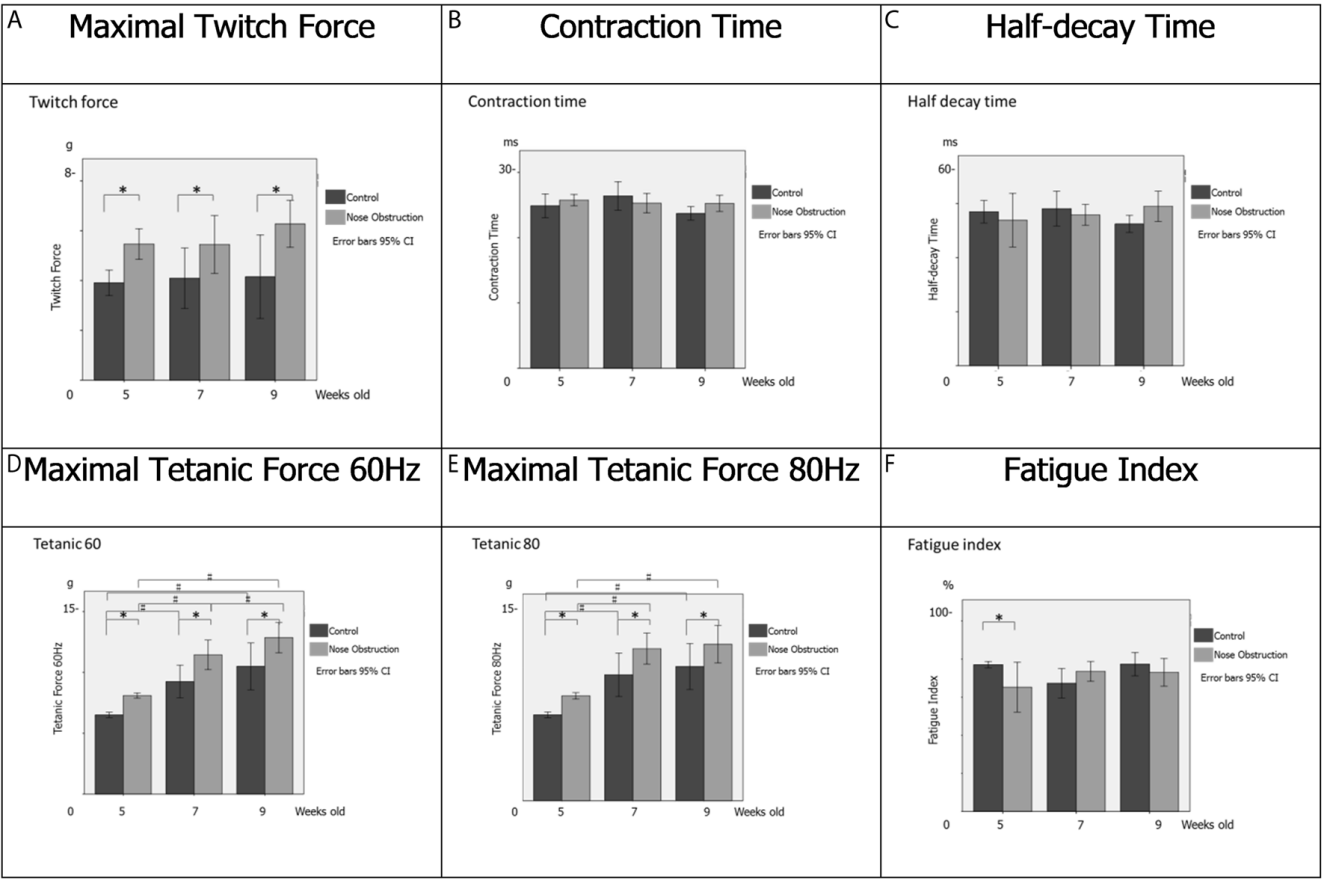
Contractile properties of Genioglossus muscle. (**A**) Maximal twitch force in grams (g) at 5, 7 and 9 weeks old. (**B**) Contraction time at 5, 7 and 9 weeks old. (**C**) Half-decay time at 5, 7 and 9 weeks old. (**D**) Tetanic force at 60 Hz at 5, 7 and 9 weeks old. (**E**) Tetanic force at 80 Hz at 5, 7 and 9 weeks old. (**F**) Fatigue index at 5, 7 and 9 weeks old. Bars in black, Control group; bars in grey, nose obstruction group. The x-axis shows the age of the rats, the y-axis shows the contractile properties measurements, for both control and nose obstruction groups. A repeated-measures multivariate analysis of variance was performed for intergroup and intragroup statistical comparisons. A simple main-effects analysis with Sidak adjustment was performed for multiple comparisons. Data are expressed as mean ± standard deviation. *^,#^*P* < 0.05. Error bars at 95% confidence interval. *5* 5 weeks old, *7* 7 weeks old, *9* 9 weeks old, *g* grams, *ms* millisecond, *%* percentage.

At 5 weeks old, there were significant differences between control and nose obstruction groups for Tw, Tet60, Tet80, and FI; while CT and HDT showed no significant difference (Table 1; Fig. 2). At 7 weeks old, Tw, Tet60, and Tet80 were significantly different, while CT and HDT were not significantly different. Similarly, at 9 weeks old, Tw, Tet60, and Tet80 were significantly different, while CT and HDT showed no significant difference.

**Table 1:** Mean values and standard deviation of the contractile properties of the genioglossus muscle.

	5 weeks old				7 weeks old				9 weeks old			
	Control		Nose obstruction		Control		Nose obstruction		Control		Nose obstruction	
	Mean	SD	Mean	SD	Mean	SD	Mean	SD	Mean	SD	Mean	SD
Tw (g)	3.9	0.6	5.5	0.7	4.1	1.5	5.5	1.4	4.2	1.8	6.3	1.0
CT (ms)	24.9	2.2	25.8	1.0	26.4	2.6	25.3	1.6	23.7	1.1	25.3	1.5
HDT (ms)	57.0	5.0	53.9	11.9	58.1	7.7	55.9	4.2	52.5	3.8	59.0	6.7
Tet60 (g)	6.5	0.3	8.1	0.2	9.3	1.6	11.5	1.5	10.5	1.6	12.9	1.5
Tet80 (g)	6.5	0.3	7.9	0.3	9.5	2.0	11.5	1.4	10.1	1.9	11.8	1.7
FI (%)	77.0	2.0	65.2	12.5	67.3	9.3	73.5	5.6	77.3	7.3	73.0	7.9

Data presented as mean mean ± standard deviation (SD).

*Tw* twitch force, *CT* contraction time, *HDT* half-decay time, *Tet60* tetanic force at 60 Hz, *Tet80* Tetanic force at 80 Hz, *FI* fatigue index, *g* grams, *ms* millisecond, *%* percentage.

There were no intragroup difference for Tw, CT, HDT and FI (Fig. 2). However, there were significant intragroup differences in Tet60 between 5- and 7-week-old animals, and 5- and 9-week-old animals in the control group; and between 5- and 7-week-old animals, 5- and 9-week-old animals, and 7- and 9-week-old animals in the nose obstruction group. The Tet80 showed significant intragroup differences between 5- and 7-week-old animals, and 5- and 9-week-old animals in both the control and the nose obstruction groups.

### Histological changes of the GG fibers

The number and percentage of slow-type and fast-type, are shown on Table 2. Fast-type muscle fibers were predominantly found in the GG, while slow-type fiber were present in low percentages in both groups at all ages. There were no significant differences between the control and nose obstruction groups at any age for either the slow or fast-type muscle fibers; and there were no intragroup statistical differences for any of these muscle fiber types.

**Table 2:** Number of slow and fast muscle fibers and percentage in genioglossus muscle.

	5 weeks old				7 weeks old				9 weeks old			
	Control		Nose obstruction		Control		Nose obstruction		Control		Nose obstruction	
	N	%	N	%	N	%	N	%	N	%	N	%
Slow	6	0.1	6	0.1	20	0.4	20	0.4	20	0.4	20	0.4
Fast	5138	99.9	5126	99.9	5135	99.6	5129	99.6	5147	99.6	5126	99.6
Total	5144	100	5132	100	5155	100	5149	100	5167	100	5146	100

Data presented as total number (N), and percentage (%).

*Slow* slow type muscle fiber, *Fast* fast type muscle fiber.

The number and percentage of fast IIa and IIb type muscle fibers are shown in Table 3. The fast-IIb type were found in greater amount than the fast-IIa type in both groups at all ages. There were no significant differences between the control and nose obstruction groups at any age for any of the IIa or IIb muscle fiber types; and there were no intragroup statistical differences for any of these muscle fiber types.

**Table 3:** Number of IIa and IIb fast genioglossus muscle fibers and percentage.

	5 weeks old				7 weeks old				9 weeks old			
	Control		Nose obstruction		Control		Nose obstruction		Control		Nose obstruction	
	N	%	N	%	N	%	N	%	N	%	N	%
IIa	347	6.70	484	9.43	520	10.09	573	11.13	527	10.20	672	13.06
IIb	2090	40.63	2166	42.21	1949	37.81	2018	39.19	1927	37.29	1935	37.60

Data presented as total number (N), and percentage (%).

*IIa* fast-twitch IIa type muscle fiber, *IIb* fast-twitch IIb type muscle fiber.

**Figure 3:**
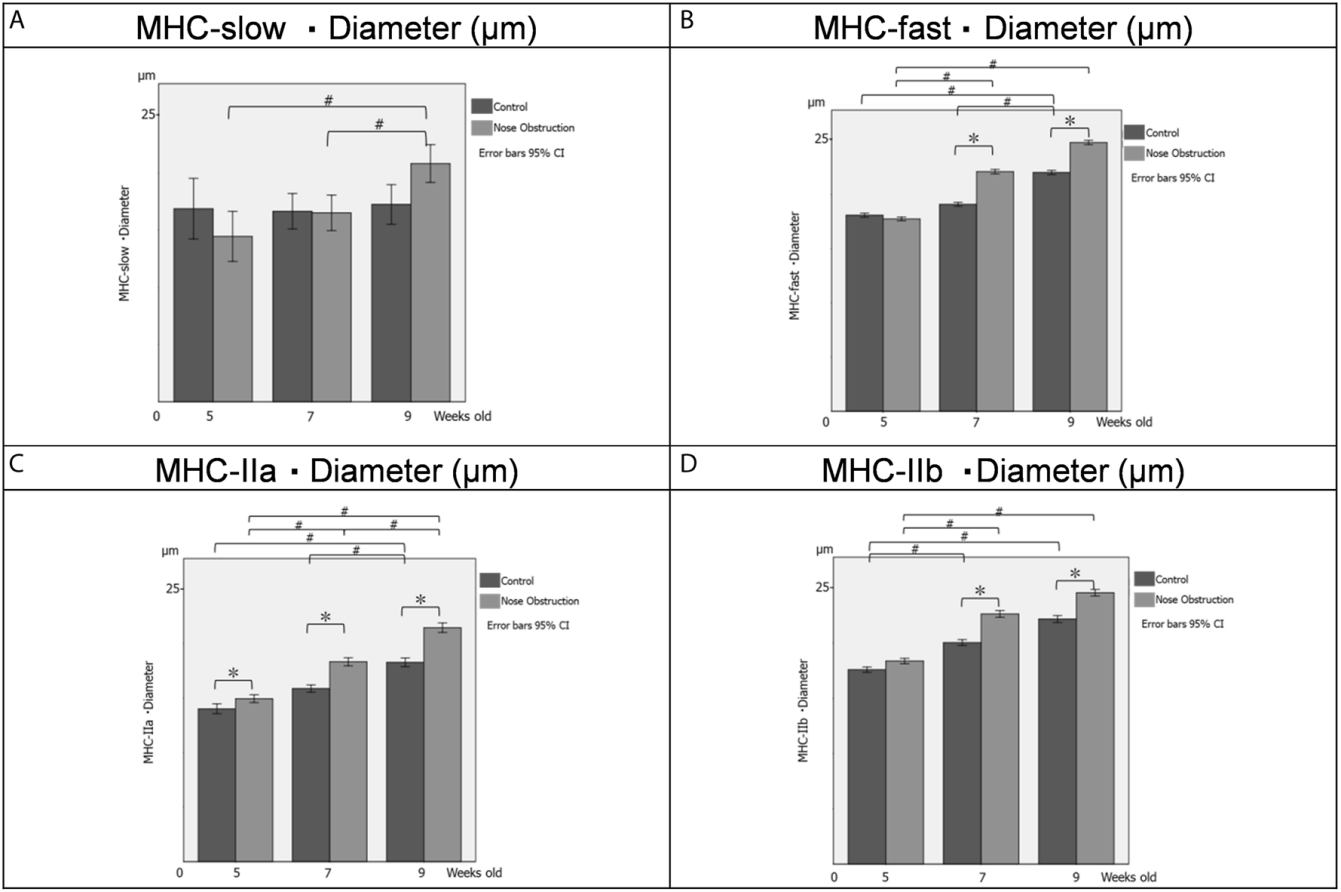
Diameter of the genioglossus muscle fibers. (**A**) Diameter of the slow-type muscle fibers at 5, 7 and 9 weeks old. (**B**) Diameter of the fast-type muscle fibers at 5, 7 and 9 weeks old. (**C**) Diameter of the fast IIa-type muscle fibers at 5, 7 and 9 weeks old. (**D**) Diameter of the fast IIb-type muscle fibers at 5, 7 and 9 weeks old. Bars in black, Control group; bars in grey, nose obstruction group. The x-axis shows the age of the rats, the y-axis shows the diameter measurement of muscle fibers, for both control and nose obstruction groups. A repeated-measures multivariate analysis of variance was performed for intergroup and intragroup statistical comparisons. A simple main-effects analysis with Sidak adjustment was performed for multiple comparisons. Data are expressed as mean ± standard deviation. *^,#^*P* < 0.05. Error bars at 95% confidence interval. *MHC-slow* Myosin heavy chain expression of slow-type muscle fiber, *MHC-fast* Myosin heavy chain expression of fast-type muscle fiber, *MHC-IIa* Myosin heavy chain expression of IIa fast-type muscle fiber, *MHC-IIb* Myosin heavy chain expression of IIb fast-type muscle fiber, *5* 5 weeks old, *7* 7 weeks old, *9* 9 weeks old, *g* grams, *ms* millisecond, *%* percentage.

At 5 weeks old, the diameter of slow-type, fast-type, and fast IIb type muscle fibers showed no significant differences between the control group and nose obstruction group, while the diameter of fast IIa type significantly increased in the nose obstruction group compared with the control group (Table 4; Fig. 3). At 7 weeks old, there were significant differences in the diameter of the fast-type, fast IIa type, and fast IIb type muscle fibers between the control and nose obstruction group, while the slow-type muscle fibers showed no significant difference in diameter between the two groups. Finally, at 9 weeks old, the diameter of the fast-type, fast IIa type, and fast IIb type muscle fibers significantly increased in the nose obstruction group compared with the control group, while the slow-type muscle fibers showed no significant difference in diameter between the two groups.

**Table 4:** Diameter values of genioglossus muscle fibers.

	5 weeks old				7 weeks old				9 weeks old			
	Control		Nose obstruction		Control		Nose obstruction		Control		Nose obstruction	
	Mean	SD	Mean	SD	Mean	SD	Mean	SD	Mean	SD	Mean	SD
Slow (µm)	16.9	4.2	14.4	3.4	16.6	3.5	16.5	3.2	17.2	3.7	20.8	3.2
Fast (µm)	18.1	6.9	17.7	6.3	19.1	6.6	22.1	7.5	22.0	7.3	24.8	7.1
IIa (µm)	14.0	4.4	14.9	4.0	15.9	3.8	18.3	4.5	18.3	4.7	21.4	5.7
IIb (µm)	17.6	5.7	18.4	5.7	20.1	6.0	22.6	7.0	22.2	7.0	24.6	6.6

Data presented as mean ± standard deviation (SD).

*Slow* slow type muscle fiber, *Fast* fast type muscle fiber, *IIa* fast-twitch IIa type muscle fiber, *IIb* fast-twitch IIb type muscle fiber.

The intragroup comparison of the diameter of the slow-type muscle fibers showed significant differences between 5- and 9-week-old rats, and 7- and 9-week-old rats in the nose obstruction group (Fig. 3). For the diameter of the fast-type fibers, there were significant differences between the 5- and 9-week-old rats, and between the 7- and 9-week-old rats in the control group, while for the nose obstruction group, there were significant differences between the 5- and 7-week-old rats, and between the 5- and 9 week-old rats. For the diameter of the fast IIa type muscle fibers, there was a significant difference between the between 5- and 9-week-old rats, and 7- and 9-week-old rats in the control group, while there were significant differences between the 5- and 7-week-old rats, the 5- and 9-week-old rats, and between the 7- and 9-week-old rats for the nose obstruction group. Finally, for the diameter of the fast IIb-type muscle fibers, there were significant differences between the 5- and 7-week-old rats, and the 5- and 9-week-old rats in the control group, while there were significant differences between the 5- and 7-week-old rats, and between the 5- and 9-week-old rats of the nose obstruction group.

**Figure 4:**
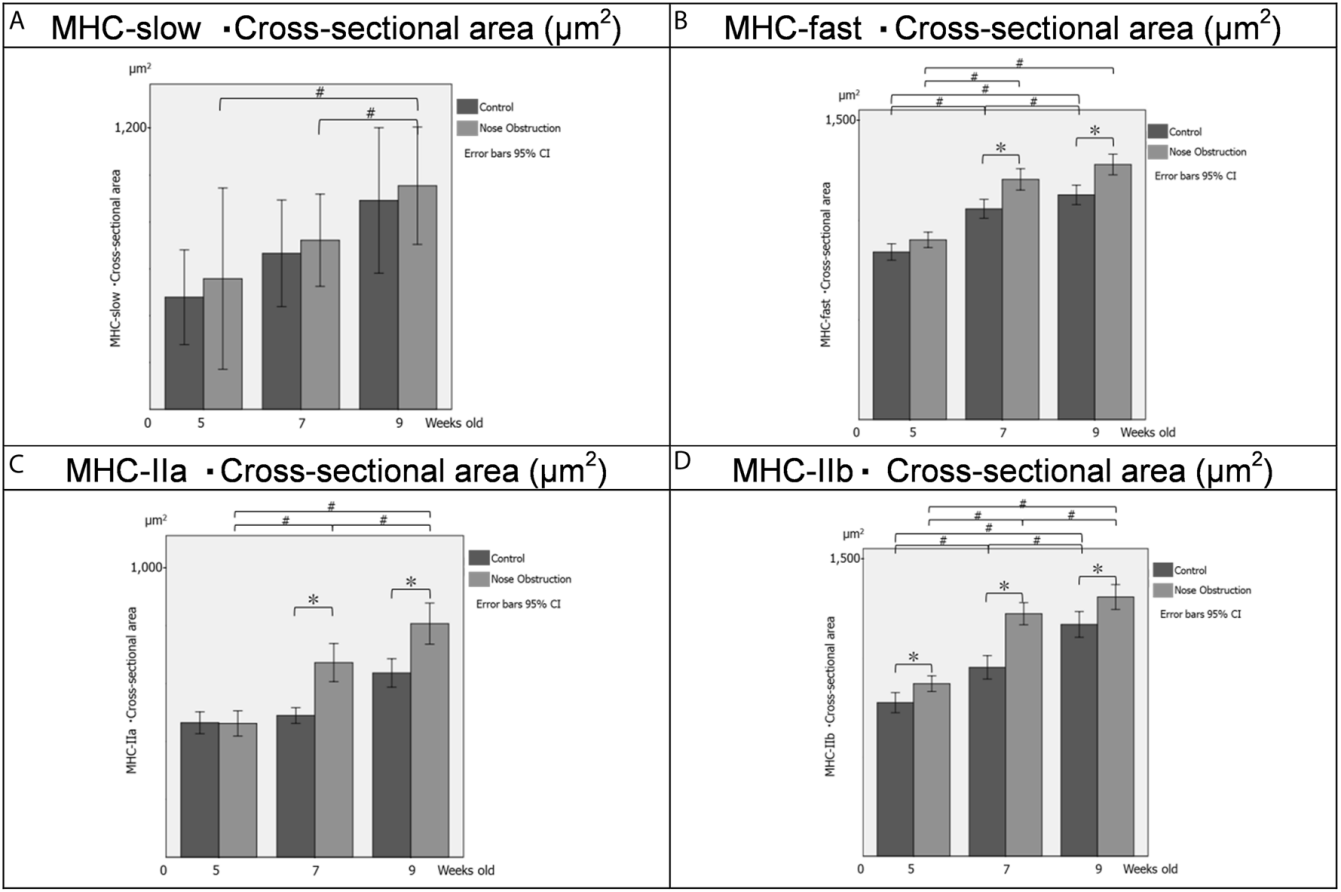
Cross-sectional area of the genioglossus muscle fibers. (**A**) Cross-sectional area of the slow-type muscle fibers at 5, 7 and 9 weeks old. (**B**) Cross-sectional area of the fast-type muscle fibers at 5, 7 and 9 weeks old. (**C**) Cross-sectional area of the fast IIa-type muscle fibers at 5, 7 and 9 weeks old. (**D**) Cross-sectional area of the fast IIb-type muscle fibers at 5, 7 and 9 weeks old. Bars in black, Control group; bars in grey, nose obstruction group. The x-axis shows the age of the rats, the y-axis shows the cross-sectional measurement of muscle fibers, for both control and nose obstruction groups. A repeated-measures multivariate analysis of variance was performed for intergroup and intragroup statistical comparisons. A simple main-effects analysis with Sidak adjustment was performed for multiple comparisons. Data are expressed as mean ± standard deviation. *^,#^*P* < 0.05. Error bars at 95% confidence interval. *MHC-slow* Myosin heavy chain expression of slow-type muscle fiber, *MHC-fast* Myosin heavy chain expression of fast-type muscle fiber, *MHC-IIa* Myosin heavy chain expression of IIa fast-type muscle fiber, *MHC-IIb* Myosin heavy chain expression of IIb fast-type muscle fiber, *5* 5 weeks-old, *7* 7 weeks-old, *9* 9 weeks-old, *g* grams, *ms* millisecond, *%* percentage.

With regards to the cross-sectional area, at 5 weeks old, there were significant differences in the cross-sectional area of the fast IIb type muscle fibers between the control group and nose obstruction group, while there were no significant differences in the cross-sectional area of the slow-type, fast-type and fast IIa type between the two groups at this age (Table 5; Fig. 4). At 7 weeks old, there were significant differences between the control and nose obstruction groups, for the fast-type, fast IIa type, and fast IIb type muscle fibers, while there were no significant difference for the slow-type muscle fiber between the control group and nose obstruction group. Likewise, at 9 weeks old, there were significant differences between the control group and nose obstruction group, for the fast-type, fast IIa type, and fast IIb type muscle fibers. On the other hand, there was no significant difference for the slow-type fibers between the two group at this age.

**Table 5:** Cross-sectional values of genioglossus muscle fibers.

	5 weeks old				7 weeks old				9 weeks old			
	Control		Nose obstruction		Control		Nose obstruction		Control		Nose obstruction	
	Mean	SD	Mean	SD	Mean	SD	Mean	SD	Mean	SD	Mean	SD
Slow (µm^2^)	478.6	192.7	558.0	243.3	666.3	339.2	722.1	275.2	892.0	295.8	955.0	326.2
Fast (µm^2^)	842.1	477.6	902.8	452.7	1058.8	575.3	1206.6	605.4	1129.0	549.9	1281.6	598.8
IIa (µm^2^)	464.5	187.6	462.2	171.7	489.6	177.8	672.3	335.5	636.7	181.8	806.9	431.6
IIb (µm^y^)	776.4	369.6	872.1	414.0	954.8	558.3	1226.0	487.5	1172.0	649.2	1310.7	625.6

Data presented as mean ± standard deviation (SD).

*Slow* slow type muscle fiber, *Fast* fast type muscle fiber, *IIa* fast-twitch IIa type muscle fiber, *IIb* fast-twitch IIb type muscle fiber.

**Figure 5:**
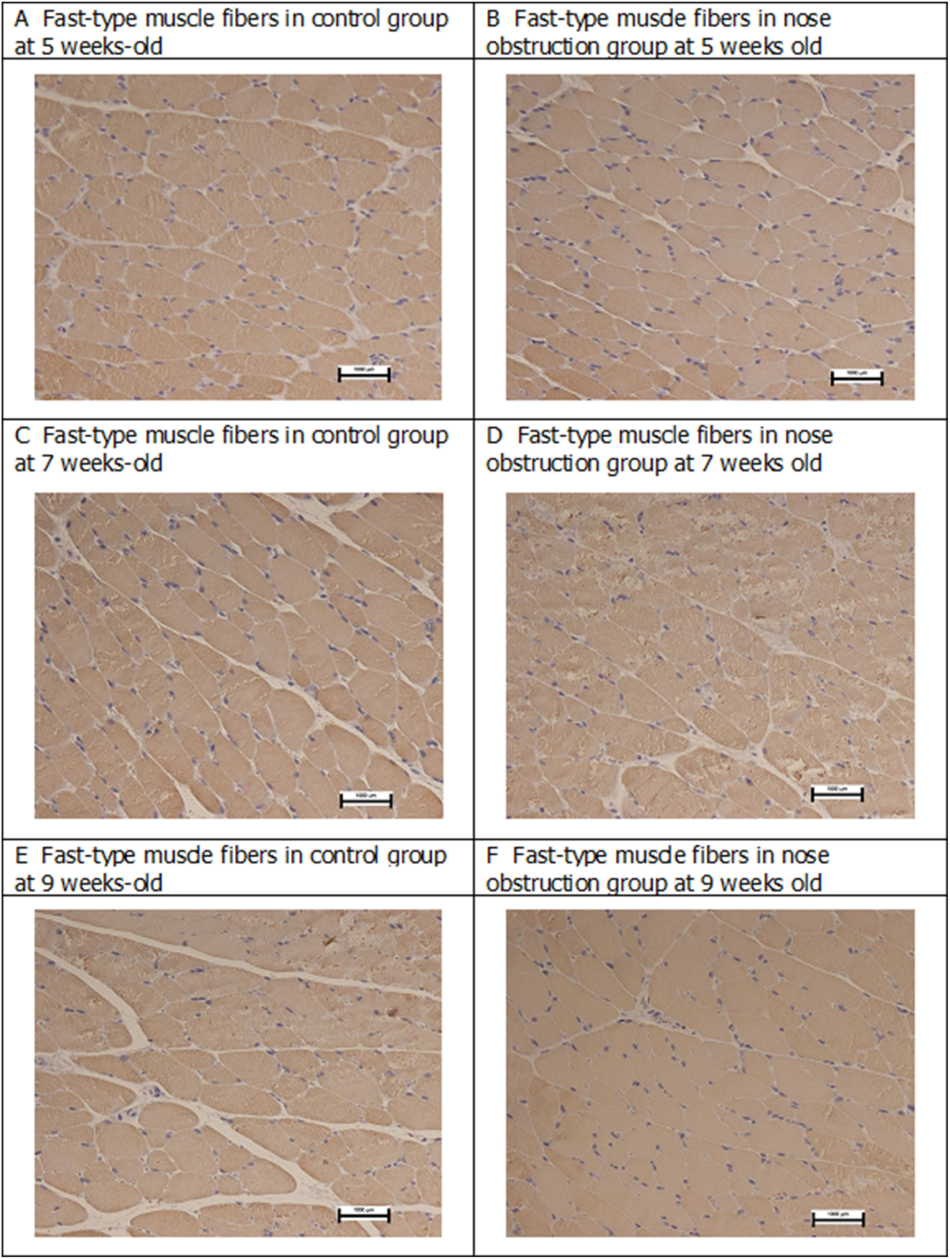
Representative images of the genioglossus fast-type muscle fibers in the 5-, 7-, and 9-week-old rats. Figures at ×20 magnitude. 1000 μm scale bar. *MHC-fast* Myosin heavy chain expression of fast-type muscle fiber.

The intragroup comparison of the cross-sectional area of the slow-type muscle fibers showed significant differences between the 5- and 9-week-old, and between the 7- and 9-week-old animals of the nose obstruction group (Fig. 4). For the fast-type cross-sectional area, the control group showed significant differences between the 5- and 7-week-old, 5- and 9-week-old, and between the 7- and 9-week old, while the nose obstruction group showed significant differences between the 5- and 7-week-old, and between the 5- and 9-week-old animals. As to the cross-sectional area of the fast IIa type fibers, there were significant differences between the 5- and 7-week-old, the 5- and 9-week-old, and between the 7- and 9-week-old animals in the nose obstruction group, while there were no intragroup differences in the control group. For the cross-sectional area of the fast IIb type muscle fibers, there were significant differences between the 5- and 7-week-old, the 5- and 9-week-old, and the 7- and 9-week-old animals in the control group. Likewise, there were significant differences between the 5- and 7-week-old, the 5- and 9-week-old, and the 7- and 9-week-old animals in the nose obstruction group. Muscle fiber samples (MHC-fast) are shown in Fig. 5.

## Discussion

The findings of this study showed that the contractile properties, diameter and cross-sectional of GG muscle fibers changed after unilateral nasal obstruction in growing rats.

### Role of the GG in respiration

The pharynx tends to collapse during inspiratory contractions if the diaphragm and accessory inspiratory muscles, as its transmural pressure becomes negative^24^. The musculature within the pharyngeal walls, or adjacent to the pharynx, dilate and/or stiffen it, such as the tongue muscles, which movement and stiffening affect the dimensions and compliance of the oropharynx and velopharynx^24^.

The GG, an extrinsic muscle of the tongue, originates from the inner surface of the lower jaw, pulling the base of the tongue down and forward when contracted, and enlarges the upper airway together with other protruding muscles^25^. GG acts as dilator of the upper airwaves by controlling the tongue’s position, moving the hyoid bone forward and upwards, stabilizing the upper airway^26^. The pressure sensors in the upper airway modulate reflexively the GG activity^25^. The forward and downward movement of the tongue, due to coactivation or independent contraction of the tongue-protruding muscles, take pressure off the soft palate, allowing it to move forward, thus dilating the pharynx^24^.

Although the rat pharynx is more rectilinear than the curved human pharynx, they have similar neural control and mechanical effects of the extrinsic tongue muscles contraction^24^. Based on the important role of the GG in the upper airway patency, changes in the breathing pattern may affect it greatly.

Our present findings suggest that indeed the contractile and histological characteristics of GG are influenced by unilateral nasal obstruction. It is possible that GG protruding force increases, by means of greater muscle fiber diameter and area, in order to help augment the diameter of the pharynx.

### Contractile and histological changes of the GG after nasal obstruction

The tongue-protruding force is increased after unilateral nasal obstruction in rats^13,14^. In this study the twitch and tetanic forces of GG increased at all ages, while the FI decreased at 5 weeks old after unilateral nasal obstruction.

The results showed the increase in the diameter and cross-sectional area of the fast-type fibers and its subforms IIa and IIb that are in correlation with the increase in both twitch force and tetanic forces.

The slow-fiber type relates to slower twitch contraction time after nerve stimulation, and the fast-type responds faster; slow-fiber type is also related to more resistance to fatigability, whereas the fast-fiber type generates more force than the slower type^16^. GG is mainly a fast-twitch muscle, containing mostly fast-type in all groups at all ages, as confirmed in our study, and this may correspond to the high performance that the tongue, and more specifically, the GG has in all the orofacial behaviours that need specifically a faster contraction response.

For nutritive suckling, the tip of the tongue protrudes against the nipple, and then retracts to exert a milking or stroking movement. In rats, the transition between suckling and chewing is between birth and postnatal day 30, and during this same period, the rat GG muscle shifts from a developmental to adult fast MHC isoform composition^27^, indicating that MHC composition adapts to the function of the tongue.

This study analysed GG muscle fibers at 5, 7 and 9 week-old, with fewer changes in the nasal obstruction 5 week-old rats, whereas the 7 and 9 week-old ages showed the most changes, due to a possible adaptation process. This, at the same time, may also reflect in the decrease in fatigability resistance showed only in the 5-week-old rats.

Changes in muscle fiber MHC composition have been previously reported^27^. Our results suggest that there is a tendency to increase the percentage of IIa-type, and decrease the IIb-type with age. It is also important to notice that IIx type is possibly the predominant type in GG between all fast-type fibers. These findings are in accordance to a previous study in young and old rats’ GG^28^, were older rats had a tendency to have more IIa-type fibers, and young rats had significantly more IIb fibers, and both groups had a predominance in IIx-type fibers. It may be possible that, as our rats are still during the growth period, more changes in the number of muscle fibers may occur. The change in the MHC composition is related to the neural control input, and requirements of the muscle.

### The GG function in the craniofacial complex

The tongue muscles act in several processes, such as suckling, respiration, mastication, swallowing and phonation^2^. The speed and precision with which the tongue must move to perform its different functions, are governed by its contractile properties, the intrinsic properties of the hypoglossal motoneurons, generator–producer rhythmic modulation of hypoglossal motoneuron activity, and stimulation from cortical and subcortical nuclei^29^. As changes in the GG contractile properties may also affect the tongue’s performance, we may suggest that unilateral nasal obstruction affects the tongue’s function in the different orofacial behaviours. It is also important to consider that nasal obstruction in rats leads to changes in the primary motor cortex^30^.

Although the rodent and human anatomical and physiological characteristics of the respiratory system are different^31^, the present animal model showed that nasal obstruction during growth would affect the GG contractile and histological properties. A previous study in humans showed that the GG activity changed with different breathing patterns, and the maximum tongue pressure was greater during oral breathing than during nasal breathing^32^. It is possible that any change from nasal to oral breathing could disrupt not only the GG function, but also its muscle fiber composition, thus affecting both the craniofacial normal development and orofacial function.

Mouth breathing patients during growth can present alterations in the normal development of the craniofacial complex, such as changes in dental position, mandibular position, facial height and palatal and maxillary constriction^33,34^. Moreover, these patients can present other orofacial functions affected, such as atypical swallowing^35^. Not only nasal breathing impairment, but other respiratory disturbances, such as obstructive sleep apnea, could present changes in the orofacial functions and muscle coordination^36^. In the obstructive sleep apnea, there is recurrent upper airway obstruction during sleep, when neuromuscular activity wanes^37^, being the GG a main factor for the airway obstruction. It should be considered that any alteration in the normal physiology and histology of the GG might influence also its normal performance and function in the different orofacial behaviours of patients with breathing problems, and predispose them to parafunctional habits involving the tongue.

Considering that muscle training exercises could affect the tongue muscle force and muscle fiber histological characteristics^38^, the findings of the current study may contribute in understanding how the GG can be affected by alteration in the breathing pattern, in order to improve the treatment approach in these conditions, utilizing techniques such as MFT. Moreover, the tongue muscles assessment could play an essential point to consider during the diagnosis and treatment of patients with different breathing disturbances, as it has been reported an improvement in the tongue motor function in patients with sleep-disordered breathing when performing a sensorimotor monitoring of the tongue muscles^10^.

### Limitations of the study

In regards with the limitations of the present study, it should be considered that the anatomical location of the genioglossus muscle present different direction of the muscle fibers, which may have been difficult to characterize in full details. This model of unilateral nasal obstruction, although alters the normal nasal breathing pattern, will not lead to actual mouth breathing, as rodents are uncapable to present a mouth breathing pattern. Moreover, the physiology and anatomy of rodents is different from humans, thus the present results, although interesting to understand more about the changes in muscle properties of the GG after nasal obstruction, may differ between species.

## Conclusion

Unilateral nasal obstruction during growth affects the rats’ GG histological and contractile properties by increasing its force, and augmenting the diameter and area of its muscle fibers.

## Data Availability

The datasets analysed during the current study are available from the corresponding author on reasonable request.

## Abbreviations

CT: Contraction time
GG: Genioglossus muscle
FI: Fatigue index
HDT: Half-decay time
MHC: Myosin-heavy chain
Tet60: Tetanic force at 60 H
Tet80: Tetanic force at 80 Hz
Tw: Twitch force

## References

[1] Bailey EF, Huang Y-H, Fregosi RF (2006). Anatomic consequences of intrinsic tongue muscle activation. J. Appl. Physiol..

[2] Chamberland M, Winter M, Brice TAW, Jones DK, Tallantyre EC (2021). Beyond lesion-load: Tractometry-based metrics for characterizing white matter lesions within fibre pathways. Math. Visual..

[3] Chamberlin NL, Eikermann M, Fassbender P, White DP, Malhotra A (2007). Genioglossus premotoneurons and the negative pressure reflex in rats. J. Physiol..

[4] Lowe AA (1980). The neural regulation of tongue movements. Prog. Neurobiol..

[5] Miller AJ (1978). Electromyography of craniofacial musculature during oral respiration in the rhesus monkey (*Macaca mulatta*). Arch. Oral Biol..

[6] Olson MD, Junna MR (2021). Hypoglossal nerve stimulation therapy for the treatment of obstructive sleep apnea. Neurotherapeutics.

[7] Pereira TC, Furlan RMMM, Motta AR (2019). Relationship between mouth breathing etiology and maximum tongue pressure. Codas.

[8] Oh JS (2021). Determinants of probable sleep bruxism in a pediatric mixed dentition population: A multivariate analysis of mouth vs. nasal breathing, tongue mobility, and tonsil size. Sleep Med..

[9] Rodríguez-Alcalá L (2022). Evaluation of the muscle strength of the tongue with the tongue digital spoon (TDS) in patients with obstructive sleep apnea. Life.

[10] Rodríguez-Alcalá L (2021). Sensorimotor tongue evaluation and rehabilitation in patients with sleep-disordered breathing: A novel approach. J. Oral Rehabil..

[11] de Felício CM, da Silva Dias FV, Trawitzki LV (2018). Obstructive sleep apnea: Focus on myofunctional therapy. Nat. Sci. Sleep.

[12] Rueda JR, Mugueta-Aguinaga I, Vilaró J, Rueda-Etxebarria M (2020). Myofunctional therapy (oropharyngeal exercises) for obstructive sleep apnoea. Cochrane Database Syst. Rev..

[13] Uchima Koecklin KH (2017). Unilateral nasal obstruction during later growth periods affects craniofacial muscles in rats. Front. Physiol..

[14] Uchima Koecklin KH (2015). Effect of unilateral nasal obstruction on tongue protrusion forces in growing rats. J. Appl. Physiol..

[15] Cullins MJ, Krekeler BN, Connor NP (2018). Differential impact of tongue exercise on intrinsic lingual muscles. Laryngoscope.

[16] MacIntosh B, Gardiner P, McComas A, Champaign I (2006). Muscle contraction. Skeletal Muscle, Form and Function.

[17] Sutlive T, Shall M, McClung J, Goldberg S (2000). Contractile properties of the tongue’s genioglossus muscle and motor units in the rat. Muscle Nerve.

[18] Sutlive TG, Mcclung JR, Goldberg SJ (1999). Whole-muscle and motor-unit contractile properties of the styloglossus muscle in rat. J. Neurophysiol..

[19] Funaki Y, Hiranuma M, Shibata M, Kokai S, Ono T (2014). Effects of nasal obstruction on maturation of the jaw-opening reflex in growing rats. Arch. Oral Biol..

[20] Nagai H, Russell JA, Jackson MA, Connor NP (2008). Effect of aging on tongue protrusion forces in rats. Dysphagia.

[21] Burke RE, Levine DN, Tsairis P, Zajac FE (1973). Physiological types and histochemical profiles in motor units of the cat gastrocnemius. J. Physiol..

[22] Connor NP, Ota F, Nagai H, Russell JA, Leverson G (2008). Differences in age-related alterations in muscle contraction properties in rat tongue and hindlimb. J. Speech Lang. Hear. Res..

[23] Mu L, Sanders I (2000). Neuromuscular specializations of the pharyngeal dilator muscles: II. Compartmentalization of the canine genioglossus muscle. Anat. Rec..

[24] Fregosi RF (2008). Influence of tongue muscle contraction and dynamic airway pressure on velopharyngeal volume in the rat. J. Appl. Physiol..

[25] Saboisky JP (2006). Tonic and phasic respiratory drives to human genioglossus motoneurons during breathing. J. Neurophysiol..

[26] Huang H (2012). Effects of chronic intermittent hypoxia on genioglossus in rats. Sleep Breath.

[27] Kinirons SA, Shall MS, McClung JR, Goldberg SJ (2003). Effect of artificial rearing on the contractile properties and myosin heavy chain isoforms of developing rat tongue musculature. J. Neurophysiol..

[28] Schaser AJ, Wang H, Volz LM, Connor NP (2011). Biochemistry of the anterior, medial, and posterior genioglossus in the aged rat. Dysphagia.

[29] Sawczuk A, Mosier KM (2001). Neural control of tongue movement with respect to respiration and swallowing. Crit. Rev. Oral Biol. Med..

[30] Abe Y (2017). Unilateral nasal obstruction affects motor representation development within the face primary motor cortex in growing rats. J. Appl. Physiol..

[31] Tsujino I, Kawakami Y, Kaneko A (2005). Comparative simulation of gas transport in airway models of rat, dog, and human. Inhal. Toxicol..

[32] Takahashi S, Ono T, Ishiwata Y, Kuroda T (1999). Effect of changes in the breathing mode and body position on tongue pressure with respiratory-related oscillations. Am. J. Orthod. Dentofac. Orthop..

[33] Basheer B, Hegde KS, Bhat SS, Umar D, Baroudi K (2014). Influence of mouth breathing on the dentofacial growth of children: A cephalometric study. J. Int. Oral Health.

[34] Lione R, Buongiorno M, Franchi L, Cozza P (2014). Evaluation of maxillary arch dimensions and palatal morphology in mouth-breathing children by using digital dental casts. Int. J. Pediatr. Otorhinolaryngol..

[35] Barata AR, Kizi G, Alves V, Proença L, Delgado A (2021). Association between mouth-breathing and atypical swallowing in young orthodontic patients at Egas Moniz Dental Clinic. Ann. Med..

[36] de Felício CM (2016). Orofacial motor functions in pediatric obstructive sleep apnea and implications for myofunctional therapy. Int. J. Pediatr. Otorhinolaryngol..

[37] Oliven A, Carmi N, Coleman R, Odeh M, Silbermann M (2001). Age-related changes in upper airway muscles morphological and oxidative properties. Exp. Gerontol..

[38] Krekeler BN, Weycker JM, Connor NP (2020). Effects of tongue exercise frequency on tongue muscle biology and swallowing physiology in a rat model. Dysphagia.

